# The dynamic surface evolution of halide perovskites induced by external energy stimulation

**DOI:** 10.1093/nsr/nwae042

**Published:** 2024-02-02

**Authors:** Feiyu Cheng, Pengdong Wang, Chenzhe Xu, Qingliang Liao, Suicai Zhang, Haochun Sun, Wenqiang Fan, Guodong Liu, Zhiyun Li, Yaping Kong, Li Wang, Fangsen Li, Zhuo Kang, Yue Zhang

**Affiliations:** Academy for Advanced Interdisciplinary Science and Technology, State Key Laboratory for Advanced Metals and Materials, University of Science and Technology Beijing, Beijing 100083, China; Beijing Key Laboratory for Advanced Energy Materials and Technologies, Key Laboratory of Advanced Materials and Devices for Post-Moore Chips, Ministry of Education, Beijing Advanced Innovation Center for Materials Genome Engineering, School of Materials Science and Engineering, University of Science and Technology Beijing, Beijing 100083, China; Vacuum Interconnected Nanotech Workstation (Nano-X), Suzhou Institute of Nano-Tech and Nano-Bionics, Chinese Academy of Sciences, Suzhou 215123, China; Academy for Advanced Interdisciplinary Science and Technology, State Key Laboratory for Advanced Metals and Materials, University of Science and Technology Beijing, Beijing 100083, China; Beijing Key Laboratory for Advanced Energy Materials and Technologies, Key Laboratory of Advanced Materials and Devices for Post-Moore Chips, Ministry of Education, Beijing Advanced Innovation Center for Materials Genome Engineering, School of Materials Science and Engineering, University of Science and Technology Beijing, Beijing 100083, China; Academy for Advanced Interdisciplinary Science and Technology, State Key Laboratory for Advanced Metals and Materials, University of Science and Technology Beijing, Beijing 100083, China; Beijing Key Laboratory for Advanced Energy Materials and Technologies, Key Laboratory of Advanced Materials and Devices for Post-Moore Chips, Ministry of Education, Beijing Advanced Innovation Center for Materials Genome Engineering, School of Materials Science and Engineering, University of Science and Technology Beijing, Beijing 100083, China; Academy for Advanced Interdisciplinary Science and Technology, State Key Laboratory for Advanced Metals and Materials, University of Science and Technology Beijing, Beijing 100083, China; Beijing Key Laboratory for Advanced Energy Materials and Technologies, Key Laboratory of Advanced Materials and Devices for Post-Moore Chips, Ministry of Education, Beijing Advanced Innovation Center for Materials Genome Engineering, School of Materials Science and Engineering, University of Science and Technology Beijing, Beijing 100083, China; Academy for Advanced Interdisciplinary Science and Technology, State Key Laboratory for Advanced Metals and Materials, University of Science and Technology Beijing, Beijing 100083, China; Beijing Key Laboratory for Advanced Energy Materials and Technologies, Key Laboratory of Advanced Materials and Devices for Post-Moore Chips, Ministry of Education, Beijing Advanced Innovation Center for Materials Genome Engineering, School of Materials Science and Engineering, University of Science and Technology Beijing, Beijing 100083, China; Academy for Advanced Interdisciplinary Science and Technology, State Key Laboratory for Advanced Metals and Materials, University of Science and Technology Beijing, Beijing 100083, China; Beijing Key Laboratory for Advanced Energy Materials and Technologies, Key Laboratory of Advanced Materials and Devices for Post-Moore Chips, Ministry of Education, Beijing Advanced Innovation Center for Materials Genome Engineering, School of Materials Science and Engineering, University of Science and Technology Beijing, Beijing 100083, China; Beijing National Laboratory for Condensed Matter Physics, Institute of Physics, Chinese Academy of Sciences, Beijing 100190, China; Vacuum Interconnected Nanotech Workstation (Nano-X), Suzhou Institute of Nano-Tech and Nano-Bionics, Chinese Academy of Sciences, Suzhou 215123, China; Vacuum Interconnected Nanotech Workstation (Nano-X), Suzhou Institute of Nano-Tech and Nano-Bionics, Chinese Academy of Sciences, Suzhou 215123, China; Vacuum Interconnected Nanotech Workstation (Nano-X), Suzhou Institute of Nano-Tech and Nano-Bionics, Chinese Academy of Sciences, Suzhou 215123, China; Vacuum Interconnected Nanotech Workstation (Nano-X), Suzhou Institute of Nano-Tech and Nano-Bionics, Chinese Academy of Sciences, Suzhou 215123, China; Academy for Advanced Interdisciplinary Science and Technology, State Key Laboratory for Advanced Metals and Materials, University of Science and Technology Beijing, Beijing 100083, China; Beijing Key Laboratory for Advanced Energy Materials and Technologies, Key Laboratory of Advanced Materials and Devices for Post-Moore Chips, Ministry of Education, Beijing Advanced Innovation Center for Materials Genome Engineering, School of Materials Science and Engineering, University of Science and Technology Beijing, Beijing 100083, China; Academy for Advanced Interdisciplinary Science and Technology, State Key Laboratory for Advanced Metals and Materials, University of Science and Technology Beijing, Beijing 100083, China; Beijing Key Laboratory for Advanced Energy Materials and Technologies, Key Laboratory of Advanced Materials and Devices for Post-Moore Chips, Ministry of Education, Beijing Advanced Innovation Center for Materials Genome Engineering, School of Materials Science and Engineering, University of Science and Technology Beijing, Beijing 100083, China

**Keywords:** metal halide perovskite, external energy stimulation, surface dynamic evolution, surface electronic structure, ultra-high vacuum interconnection system

## Abstract

Tracking the dynamic surface evolution of metal halide perovskite is crucial for understanding the corresponding fundamental principles of photoelectric properties and intrinsic instability. However, due to the volatility elements and soft lattice nature of perovskites, several important dynamic behaviors remain unclear. Here, an ultra-high vacuum (UHV) interconnection system integrated by surface-sensitive probing techniques has been developed to investigate the freshly cleaved surface of CH_3_NH_3_PbBr_3_  *in situ* under given energy stimulation. On this basis, the detailed three-step chemical decomposition pathway of perovskites has been clarified. Meanwhile, the evolution of crystal structure from cubic phase to tetragonal phase on the perovskite surface has been revealed under energy stimulation. Accompanied by chemical composition and crystal structure evolution, electronic structure changes including energy level position, hole effective mass, and Rashba splitting have also been accurately determined. These findings provide a clear perspective on the physical origin of optoelectronic properties and the decomposition mechanism of perovskites.

## Teaser

By developing an integrated ultra-high vacuum interconnect system with surface-sensitive detection technology, the dynamic evolution of perovskite surfaces under external energy stimulation has been deciphered. This encompasses insights into chemical degradation pathways, crystal, and electronic structure evolution, paving the way for designing and synthesizing highly stable halide perovskite materials.

## INTRODUCTION

Metal halide perovskites (MHPs) are predicted to be ideal candidates for next-generation photovoltaic materials due to their low fabrication cost and remarkable photoelectric features such as adjustable optical band gap, high absorption coefficient, long diffusion length of carriers, and high defect tolerance [1–6]. However, the poor long-term stability of MHPs is the main hindrance to their commercial application. Among the factors leading to MHP decomposition, external energy stimulation is the major barrier to perovskites’ widespread use [7]. Furthermore, perovskite materials exhibit a complex dynamical system that results in unusual dynamical behavior under external stimulation [8], making it difficult to characterize their structure and properties precisely [9]. The detailed degradation pathway, hidden structural dynamics, and evolution of electronic structure in perovskite materials under external stimulation are still unclear [9–11]. It is critical to accurately describe the dynamic evolution of perovskite to rationally design perovskite structures and regulate their properties.

The surface of perovskite is not only important for its photoelectric properties and stable behavior but also important for understanding its inherent properties [12–14]. Understanding the dynamic evolution of surfaces is fundamental to resolving the controversial issue of perovskite materials and perovskite photovoltaics. However, hidden microstructures and underdetermined effects are difficult to detect under external stimulation due to their volatile chemical composition and soft lattice structure [15–18]. Because of a highly volatile element, MHPs undergo irreversible decomposition under external stimulation, which alters the surface composition and morphology [19,20]. Moreover, due to the soft lattice properties and liquid-like surfaces of MHPs, external stimulation will cause dynamic structural fluctuations, like lattice strain and phase transition [21–24]. Surface electronic structures, such as energy level locations, effective masses, and the Rashba splitting effect, will also evolve along with surface chemical composition and crystal structure [12]. Therefore, in order to gain a deeper understanding of the dynamic evolution of MHPs under external stimulation, all these hidden evolution details need to be considered. The key to unlocking hidden evolution details lies in the rational development of *in-situ* characterization techniques and their interpenetration with theoretical guidance.

The vacuum interconnected system links the individual equipment by an ultra-high vacuum (UHV) tube, and the surface information can be *in situ* characterized by a variety of surface analysis equipment, allowing a systematic investigation of the dynamic evolution of perovskite surfaces under external stimulation from different perspectives. UHV conditions can prevent other environmental factors from affecting perovskite surfaces. Among all surface characterization techniques, the photoelectron spectroscopic (PES) technique is one of the most important analytical methods to reveal the elemental composition, lattice structure, electronic (band) structure, physical and chemical information of halide perovskites [25,26]. Moreover, PES equipment with its radiation source can stimulate and age the perovskite surface during the measurement process without introducing additional energy stimulation, avoiding multiple external energy-induced interferences [20,27]. Thus, the combination of PES and vacuum interconnection system provides an ideal environment for revealing the dynamic surface evolution of perovskites induced by external energy stimulation.

In this work, we develop a UHV interconnection system integrated by surface-sensitive spectroscopic techniques such as angular dependent X-ray photoelectron spectroscopies (ADXPS) and angle-resolved photoelectron spectroscopy (ARPES) to *in-situ* investigate the freshly cleaved surface of CH_3_NH_3_PbBr_3_. The radiation source of the photoelectron spectroscopic equipment is designed to stimulate and age samples, avoiding the influence of external energy sources from the root. The detailed degradation processes on perovskite surfaces are determined by ADXPS as follows: the C−N bonds of CH_3_NH_3_^+^(MA^+^) cations are first broken and released NH_3_; and then the surface perovskite structure collapses, converting its Pb^2+^ into metallic Pb^0^; finally, the degradation rate slows down and the surface becomes relatively stable. Meanwhile, the evolution of the crystal structure from cubic phase to tetragonal phase on the perovskite surface is revealed by ARPES results combined with density functional theory (DFT) calculations. Following the structural evolution of perovskites, electronic structure evolution under external stimulation including energy level downward bend and hole effective mass determination is also analyzed in detail. Furthermore, the origin of Rashba splitting on the surface is explained. These findings provide valuable details of the dynamic evolution of the perovskite surface under external stimulation and enhance our comprehensive understanding of the photoelectric properties and failure mechanism of MHPs.

## RESULTS AND DISCUSSION

**Figure 1.**
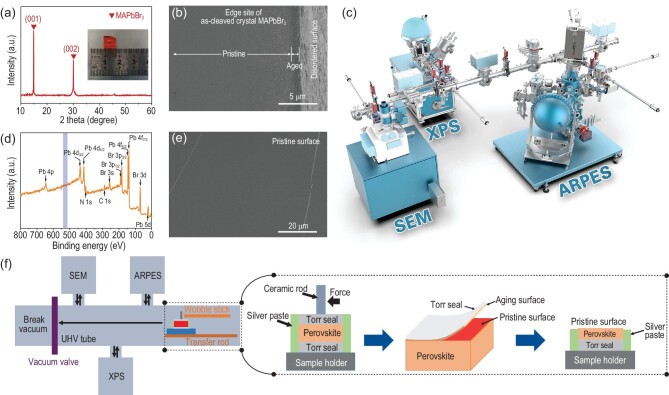
*In-situ* cleavage of MAPbBr_3_ single crystal surfaces in UHV conditions. (a) The powder XRD patterns of MAPbBr_3_ single crystals with a clear cubic structure, and the inset shows the measured photograph of MAPbBr_3_ single crystal. (b) Cross-sectional SEM of MAPbBr_3_ single crystals. (c) Schematic illustration of vacuum interconnected system for *in-situ* characterization of MAPbBr_3_ single crystal surfaces. (d) Full XPS spectra of MAPbBr_3_ pristine surface cleaved under UHV conditions. (e) SEM image showing the smooth surface of the as-cleaved single crystal. (f) Schematic diagram of samples cleavage *in-situ* under UHV conditions.

CH_3_NH_3_PbBr_3_ (MAPbBr_3_) is chosen because of its good air stability and easy cleavage along (001) [28]. The as-measured single crystals of MAPbBr_3_ are millimeter scale and large enough for XPS and ARPES measurements, as shown in the inset of Fig. 1a. The XRD curves of the crystals MAPbBr_3_ shown in Fig. 1a reveal that the MAPbBr_3_ crystals exhibit high intensity along the (001) and (002) facets, which is well matched with the cubic phase (*Pm*$\bar{3}$*m*). The lattice parameters can be confirmed as 5.92 Å, which is in good agreement with previous research [29]. There are no irrelevant peaks in the XRD pattern, indicating a pure cubic phase of MAPbBr_3_. The cross-sectional SEM image shows the contrast in morphologies between pristine and aged edge sites on the MAPbBr_3_ crystals in Fig. 1b. The dark area is the crystal surface which transforms and becomes disordered due to water and oxygen in the air [30]. However, when the surface is cleaved in the air condition, moisture and oxygen are rapidly attracted to the pristine surface, forming the hydrate that will still mask the natural properties of the surface, as depicted in Figs S1 and S2. To conduct further experiments, a clean and smooth surface is required.

Figure 1f illustrates schematically how samples are cleaved along the (001) surface of crystals under ultra-high vacuum conditions, and the detailed cleavage processes are described in the Supplementary Information. Figure 1d is the XPS full spectra of the pristine crystal surface cleaved under UHV conditions without O element peaks, and there is no peak attributed to the O 1s core-level spectra in Fig. S1. The results indicate that the surface cleaved *in situ* under UHV conditions is very clean and without water or oxygen erosion. The SEM image presented in Fig. 1e shows the pristine surface is very smooth on a large scale. With the higher magnification shown in Fig. S2, the SEM images of the cleaved perovskite under UHV conditions still demonstrate a smooth and flat surface. Therefore, the pristine surface obtained in this way is very flat and neat. Surface information is measured *in situ* (Fig. 1c) in the vacuum interconnection system, and the UHV environment prevents the surface from uncontrollable changes induced by ambient air.

**Figure 2.**
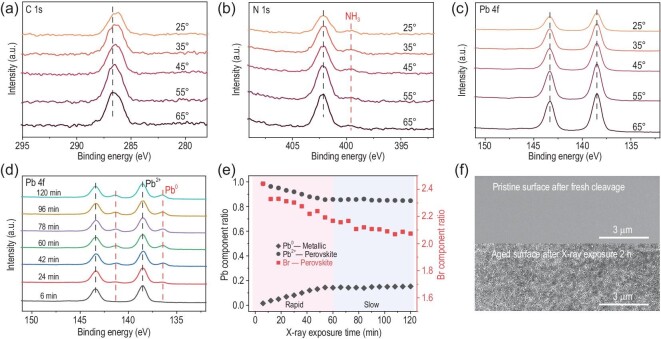
ADXPS and continuous X-ray irradiation XPS measurements of MAPbBr_3_ single crystals for chemical degradation analysis. ADXPS spectra of (a) C 1s, (b) N 1s and (c) Pb 4f core levels obtained for pristine MAPbBr_3_ at different emission angles *θ. θ* is defined as between the collected photoelectron and the surface. (d) XPS core-level spectra evolution of Pb 4f with increasing X-ray exposure time. (e) The component ratio of perovskite Pb to metallic Pb and Br/Pb with increasing X-ray irradiation time. (f) The SEM images of the surface of a freshly cleaved and after X-ray exposure for 2 h. All the spectra are calibrated to 138.5 eV, corresponding to a Pb 4f_7/2_ peak.

To explore the initial evolution of perovskite surface chemical elements, ADXPS measurements were employed. The detection depth of ADXPS depends on the emission angles (*θ*) with about *3λsinθ*, here *λ* is the inelastic mean free path of photoelectrons, and *θ* is between the collected photoelectron and the surface [31]. Thus, XPS sampling depth can be adjusted according to the emission angles (*θ*), and Table S1 illustrates the accurate sampling depths of each element of MAPbBr_3_ at detection angles between 25° and 65°. Figure 2 shows the evolutions of C 1s, N 1s, and Pb 4f core-level spectra of crystal MAPbBr_3_ (Fig. 2a–c) at different emission angles (*θ*). Figure 2a shows no adventitious carbon peaks, further proving that the as-cleaved sample is contamination-free. The C 1s peak shifts toward lower energy with decreasing emission angle, suggesting a chemical environment change for MA cations. As shown in Fig. 2b, there are two peaks at 402.2 eV and 399.5 eV for the N 1s core-level spectra, one at higher binding energy is referred to the C−N bond due to MA^+^, and the other peak at lower binding energy can be attributed to NH_3_. This is due to the fact that cation MA^+^ decomposes into NH_3_ and a hydrocarbon complex −CH_2_− under external energy stimulation, while NH_3_ escapes and leaves −CH_2_− on the surface of the sample [19]. As is shown in Fig. S3, the NH_3_/Pb ratio decreased from 0.28 to 0.13 with emission angles increased from 25° to 65°, indicating that NH_3_ adsorbs on the surface and does not exist in the bulk. The Pb 4f ADXPS spectra of the MAPbBr_3_ surface in Fig. 2c show no distinguishable changes and no additional peaks with different emission angles. There are also no changes in Br 3d peaks in Fig. S4 under different emission angles, and the Br/Pb ratios at different emission angles are nearly unchanged and close to nominal stoichiometry (Fig. S3). The ADXPS results strongly suggest that initial degradation takes place mainly in organic cations.

To explore the subsequent surface degradation processes under external energy stimulation, X-ray aging measurement research was conducted in which the sample was continually exposed to the probing X-ray resource during XPS measurements. As shown in Fig. 2d, the new metallic Pb peak occurs at 136.6 eV (Pb^0^ 4f_7/2_) and 141.4 eV (Pb^0^ 4f_5/2_) under X-ray irradiation. It is widely known that MAPbBr_3_ will first break down into PbBr_2_ and other organic components. PbBr_2_ is extremely unstable when exposed to light and X-rays, and Pb^0^ is a photodegradation byproduct of PbBr_2_ [27]. The intensities of metallic Pb peaks become stronger with the increase in X-ray exposure time, and the ratios of perovskite Pb^2+^ (138.5 eV) with metallic Pb^0^ (136.6 eV) are depicted in Fig. 2e. As X-ray irradiation time increases, more perovskite Pb^2+^ is converted into metallic Pb^0^, and the content of Br element is significantly reduced. The ratios of C/Pb and N/Pb decrease with increasing exposure time and can be seen in Fig. S5. However, the metallic Pb ratio is still less than 20% under 2 h X-ray irradiation, and the Br concentration seemed to level out after around 1 h of exposure [20]. These results indicate that sub-stable structures are present on the surface after a period of irradiation.

Following the XPS measurements, the samples were transferred to the SEM chamber by the UHV interconnected system. Figure 2f shows the SEM images after X-ray exposure for 2 h. Compared to the freshly cleaved surface, the surface exposed to X-ray becomes roughened, creating a lot of cracks and pinholes. The change in morphology also demonstrates that the perovskite surface has undergone significant surface decomposition after X-ray irradiation. To confirm that the surface degradation is originated from X-ray irradiation during XPS measurements rather than the UHV condition, the sample was placed in a vacuum for comparison. As shown in Fig. S6, only a very small amount of metallic Pb^0^ was observed in the vacuum control samples, and the SEM morphologies of the MAPbBr_3_ surface in Fig. S7 also show slight changes in the dark after 2 h of vacuum. These results imply that the stability of perovskite is also affected by vacuum conditions, as previously reported [20]. However, compared with X-ray irradiation conditions, the amount of metal lead induced under vacuum exposure conditions is significantly less (Fig. S6), and the surface morphology is flatter and more uniform (Fig. S7). Therefore, the external energy stimulation of X-ray is the primary factor in the observed degradation process, rather than the UHV conditions. The evolution of the perovskite surface is also influenced unequally by different energy stimulations and environmental conditions [32–34]. Combining the ADXPS and continuously probing X-ray experiment results, a three-step decomposition process is defined during the XPS measurement. The surface cations MA^+^ are first broken into the NH_3_ and hydrocarbon complex −CH_2_−, then the collapse of the perovskite framework into PbBr_2_, and the PbBr_2_ is further degraded into metallic Pb^0^, finally, the surface of the perovskite tends to be stable and its decomposition slows down.

**Figure 3.**
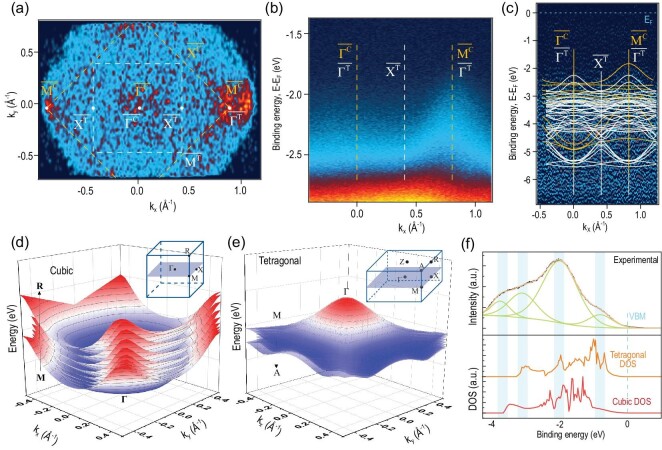
ARPES measurements and DFT calculations of MAPbBr_3_ surface electronic structure for phase transition analysis. (a) ARPES experimental constant energy cutting of the electronic structure at valence band maximum (VBM) of crystals MAPbBr_3_. Cubic and tetragonal surface Brillouin zone are shown in yellow and white, and C and T represent the cubic and tetragonal phase, respectively. (b) Top valence band dispersion map along the cubic $\overline {{\mathrm{\Gamma^ C}}} \ \overline {{\mathrm{M^C}}} $ (tetragonal $\overline {{\mathrm{\Gamma^ T}}} \ \overline {{\mathrm{X^T}}} \ \overline {{\mathrm{\Gamma^ T}}} $) direction. (c) Second derivative dispersion of MAPbBr_3_ along $\overline {{\mathrm{\Gamma^ C}}} \ \overline {{\mathrm{M^C}}} $ ($\overline {{\mathrm{\Gamma^ T}}} \ \overline {{\mathrm{X^T}}} \ \overline {{\mathrm{\Gamma^ T}}} $) direction. The calculated band dispersions based on the cubic and tetragonal phase of MAPbBr_3_ are given with yellow and white lines. (d and e) The calculated three-dimensional valence band structures of (d) cubic and (e) tetragonal phases of MAPbBr_3_. The insets of panels (d) and (e) show the measured Brillouin zone (BZ) in bulk BZ of a cubic and tetragonal phase, respectively. (f) ARPES photoemission intensity is integrated along the $\overline {{\mathrm{\Gamma^ C}}} \ \overline {{\mathrm{M^C}}} $ ($\overline {{\mathrm{\Gamma^ T}}} \ \overline {{\mathrm{X^T}}} \ \overline {{\mathrm{\Gamma^ T}}} $) direction and compared to total DOS calculations for cubic and tetragonal phase valence bands.

Further investigation of surface lattice variations of MAPbBr_3_ single crystals is conducted using ARPES. With the ARPES technique, we can measure the kinetic energy and momentum distribution on the surface of the sample, revealing the inherent charge transfer characteristics of perovskite and inferring changes in surface lattice structure. Figure 3a shows the ARPES data for the constant energy cut of the electronic structure at the valence band maximum (VBM) of MAPbBr_3_ crystals. It should be noted that in our experiments, ARPES measurement can only cover the surface Brillouin zone (BZ) ${\mathrm{\overline{\Gamma\, }}}$ ${\mathrm{\overline{M}}}$ ${\mathrm{\overline{X}}}$ plane which is the electron wave vector component parallel to the surface (*k_x_* and *k_y_*), while the surface-perpendicular component of the electron wave vector (*k_z_*) is dependent on the photoelectron kinetic energy [35]. From the calculated three-dimensional valence band structure of the cubic phase of MAPbBr_3_ in Fig. 3c, it is clear that the $\overline {{\mathrm{M^C}}} $ points are the maximum of the valence band in the cubic phase surface BZ. The highest intensity at the valence band maximum allows us to identify the $\overline {{\mathrm{M^C}}} $ point in ARPES results, therefore the surface cubic BZ is confirmed and highlighted as yellow squares in Fig. 3a. The size of the first BZ can be recognized at ∼1 Å^−1^, which agrees well with the lattice parameters of cubic MAPbBr_3_ obtained by XRD results above (*Δk_X__−Γ−X_* = 2π/*a*, where *Δk_X__−Γ−X_* is the Γ−X distance of the first BZ, *a* is the lattice parameter), and the bulk cubic BZ is shown in the inset of Fig. 3c. When the binding energy is deeper, the intensity at the X point is higher, and the ARPES experimental result is displayed in Fig. S8. Based on the theoretical calculation results in Fig. 3c, the top VB of the cubic phase at the $\overline {{\mathrm{\Gamma^ C}}} $ (0 0 0) point should be the lowest point. However, there is additional band dispersion at the $\overline {{\mathrm{\Gamma^ C}}} $ point from ARPES experimental results in Fig. 3a, and this cannot explain the cubic phase band structure. Figure 3b clearly shows the additional dispersion along the $\overline {{\mathrm{\Gamma^ C}}} \ \overline {{\mathrm{M^C}}} $ direction. The top valence band is at the $\overline {{\mathrm{M^C}}} $ point, and near the $\overline {{\mathrm{\Gamma^ C}}} $ point, the valence band dispersion is relatively flat, but the experimental band dispersion of the $\overline {{\mathrm{\Gamma^ C}}} $ point is higher. In Fig. S9, the 2D curvature band map makes it easier to notice the reproducible band dispersions at the $\overline {{\mathrm{\Gamma^ C}}} $ point. The calculations about the tetragonal phase predict the VBM at the $\overline {{\mathrm{\Gamma^ T}}} $ (0 0 0) point from Fig. 3e, and the tetragonal bulk BZ is shown in the inset of Fig. 3e. The additional dispersion at the $\overline {{\mathrm{\Gamma^ T}}} $ (0 0 0) point is most likely a result of the contribution of a tetragonal crystal phase and the surface BZ is illustrated in Fig. 3a with white squares. The high symmetry points are shown in Fig. 3a and b, where the $\overline {{\mathrm{\Gamma^ C}}} \ \overline {{\mathrm{M^C}}} $ direction in the cubic phase changes to $\overline {{\mathrm{\Gamma^ T}}} \ \overline {{\mathrm{X^T}}} \ \overline {{\mathrm{\Gamma^ T}}} $ in the tetragonal phase.

Figure 3c shows *k*-space 2D curvature band maps along the $\overline {{\mathrm{\Gamma^ C}}} \ \overline {{\mathrm{M^C}}} $ ($\overline {{\mathrm{\Gamma^ T}}} \ \overline {{\mathrm{X^T}}} \ \overline {{\mathrm{\Gamma^ T}}} $) direction, and the valence band calculated is also added to Fig. 3c for comparison. The experimental top valence band at $\overline {{\mathrm{M^C}}} $ point agrees well with the DFT calculations of cubic-phase MAPbBr_3_ (the yellow line in the plots), and the top valence band dispersions at ${\mathrm{\overline{\Gamma }}}\ $point corresponds to the calculated band structure of the tetragonal phase along the $\overline {{\mathrm{\Gamma^ T}}} \ \overline {{\mathrm{X^T}}} $ direction (the white line in the plots). The dense bands at the deeper band energy are likely due to the superposition of tetragonal and cubic valence bands. As demonstrated in Fig. 3f, the ‘experimental density of states (DOS)’ is calculated by integrating the photoemission intensity of ARPES spectra along the $\overline {{\mathrm{\Gamma^ C}}} \ \overline {{\mathrm{M^C}}} $ direction, which is compared with the calculated total DOS of the tetragonal and cubic phases. It can be found that the ‘experimental DOS’ agrees with the combined tetragonal and cubic DOS. These results indicate that there is a tetragonal phase besides the original cubic phase on the surface.

Due to the polar and soft fluctuations of MHP lattices [22], reversible dynamic lattice responses such as lattice expansion and octahedral tilt easily occur under external stimulation or perturbation [21,28]. Surface defects formed under external stimulation can reduce the activation energy of phase transition and increase the phase propagation rate [23]. Throughout the ARPES measurements, UV light (21.2 eV by He Ⅰα lamp) is always probing the sample, which aids the perovskite surface to overcome the kinetic energy barrier for the phase transition [36]. After the ARPES measurements, the samples were immediately transferred to the XPS chamber by the UHV interconnected system. The XPS results are similar to the continuously probing X-ray experiment in Fig. S10, suggesting that surface degradation and phase transition take place simultaneously during spectroscopic experiments. The dynamic evolution of perovskite surface induced by external energy stimulation, including chemical degradation and phase transition, is schematically shown in Fig. S11.

**Figure 4.**
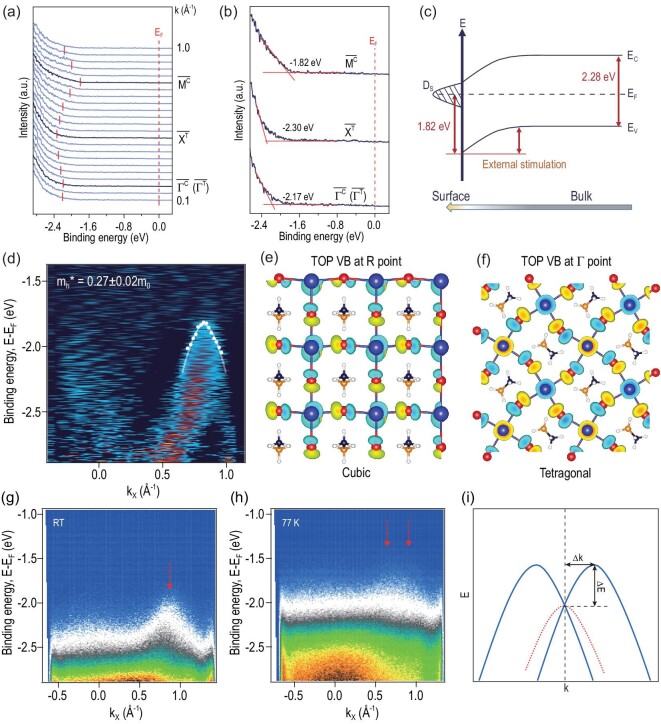
Determination of the surface electronic structure of MAPbBr_3_ after chemical degradation and phase transition. (a) Energy distribution curves (EDCs) along the $\overline {{\mathrm{\Gamma^ C}}} \ \overline {{\mathrm{M^C}}} $ ($\overline {{\mathrm{\Gamma^ T}}} \ \overline {{\mathrm{X^T}}} \ \overline {{\mathrm{\Gamma^ T}}} $) direction. The VB peak locations at different ${k}_\parallel $ are shown by marks on the EDCs, and the high symmetry momentum points${\mathrm{\ }}\overline {{\mathrm{M^C}}} $, $\overline {{\mathrm{X^T}}} $, and ${\mathrm{\overline{\Gamma }}}$ are marked in bold. (b) Determine the change in VB peak position at different high symmetry points. (c) Schematic diagram of the energy band, in which surface trap states induce downward surface band bending from bulk to surface. D_s_: surface density states of traps. (d) Second derivative dispersion maps and parabolic band fitted around the band maximum. White dots represent VB peaks. (e and f) A real space representation of the top VB wave functions of (e) a cubic MAPbBr_3_ single crystal near the R point, and (f) a tetragonal phase near the Γ point. (g and h) Valence band dispersion of MAPbBr_3_ along the $\overline {{\mathrm{\Gamma^ C}}} \ \overline {{\mathrm{M^C}}} $ ($\overline {{\mathrm{\Gamma^ T}}} \ \overline {{\mathrm{X^T}}} \ \overline {{\mathrm{\Gamma^ T}}} $) direction (g) at room temperature and (h) at liquid nitrogen temperature (77 K). The VB dispersion at 77 K shows confirmed Rashba-type splitting. (i) A schematic of Rashba-type effect on valence band dispersion.

Moreover, the electrical band structure, including the band gap and effective mass at the band gap edge, can be significantly affected by chemical degradation and phase transition on the perovskite surface. As shown in Fig. 4a and b, the energy distribution curves (EDCs) (photoemission intensity as a function of electron binding energy for defined emission angles at different ${k}_\parallel $ values) are drawn along the $\overline {{\mathrm{\Gamma^ C}}} \ \overline {{\mathrm{M^C}}} $ ($\overline {{\mathrm{\Gamma^ T}}} \ \overline {{\mathrm{X^T}}} \ \overline {{\mathrm{\Gamma^ T}}} $) direction for detailed analysis of the valence band change. In Fig. 4a, the valence band edge locations could be acquired and marked at each direction of ${k}_\parallel $ by the red line. As mentioned above, VBM is located at the $\overline {{\mathrm{M^C}}} $ point, and as the emission angle decreases, the peak gradually shifts toward the lower binding energy until reaching the $\overline {{\mathrm{X^T}}} $ point. When the diffraction angle continues to decrease, the peak continues to move higher again. Figure 4b shows the high symmetry direction $\overline {{\mathrm{M^C}}} $, $\overline {{\mathrm{X^T}}} $, ${\mathrm{\overline{\Gamma }}}$. The binding edge of this state changes from about −1.82 eV at $\overline {{\mathrm{M^C}}} $ point (${k}_\parallel $ ≈ 0.75 Å^−1^) to about −2.30 eV at $\overline {{\mathrm{X^T}}} $ point (${k}_\parallel $ ≈ 0.37 Å^−1^) and to about −2.17 eV at ${\mathrm{\overline{\Gamma }}}$ point (${k}_\parallel $ = 0 Å^−1^). The valence band maximum (VBM) is located at binding energy E−E_F_ = −1.82 eV. Based on the absorption spectra and Tauc plot in Fig. S12, the bandgap of MAPbBr_3_ is 2.28 eV. It can be seen that the Fermi level E_F_ is very much closer to the conduction band than the valence band, suggesting that the MAPbBr_3_ surface is n-type. This is mainly due to the surface degradation mentioned in the above XPS results, where metallic Pb^0^ exists on the surface with donor levels. As depicted in Fig. 4c, under external stimulation, the surface trap states represented by metallic Pb^0^ cause downward surface band bending from bulk to surface.

Furthermore, the hole effective mass m_h_* can be derived from ARPES measurements. Figure 4d shows the ARPES data and corresponding parabola fitting around the band maximum. The obtained m_h_* is 0.27 ± 0.02 *m_0_* at $\overline {{\mathrm{M^C}}} $ point for the MAPbBr_3_, where *m_0_* is the free electron mass. In light of our DFT calculation (Fig. S13), the hole effective mass m_h_* was 0.34 *m_0_* for the cubic phase at M point and 0.24 *m_0_* for the tetragonal phase at Γ point. The experimental result m_h_* is between theoretical calculations for cubic and tetragonal phases. This is also consistent with the above analysis of phase transition on the MAPbBr_3_ surface. The wave function at VBM of cubic and tetragonal surface (001) are illustrated in Fig. 4e and f. Based on these diagrams, the VBM of MAPbBr_3_ is mainly contributed by the orbitals of the Pb and Br atoms. Thus, the evolution of the top VB mainly results from the atomic displacement of Br and Pb induced by phase transition under external stimulation, and the stretching and bending of Pb-Br bonds could have a significant effect on effective masses [37]. As shown in Fig. 4e and f, the organic cations provide a negligible wave function to the VBM, and the partial density of states (PDOS) in Fig. S14 also indicates that the cation MA has a negligible influence on the DOS of the top VB. Though the MA cations cannot directly affect the top VB, the MA vacancy after surface decomposition can cause Br atom rearrangement and variations in the top VB [38].

As is well known, the strong spin orbit coupling (SOC) of perovskite may produce the Rashba effect in the presence of a local electric field with reversal symmetry violation [24,39]. The Rashba-type effect has been proposed to explain the remarkable properties of perovskites [39,40]. The Rashba effect can cause a splitting band into two bands by lifting the spin degeneracy in *k*-space by Δ*k* and shifting the valence band maxima of depth ΔE away from the high symmetry directions, and the schematic is presented in Fig. 4i. However, the Rashba splitting is not observed in ARPES results at room temperature (Fig. 4g), despite the MHPs undergoing a transition from cubic to tetragonal phase under external stimulation, which may be due to the very small Rashba effect induced by surface reconstruction [41]. Nevertheless, when the sample temperature drops to liquid nitrogen temperature (77 K), the larger Rashba-type splitting is observed from the ARPES result (Fig. 4h). Compared with the situation at room temperature, the rise of valence band energy and the shift in *k* momentum can be clearly observed. The ARPES results show that the Rashba-type splitting is greater at low temperatures because the perovskite framework of the [PbBr_6_]^4−^ octahedron rotates rigidly at low temperatures, and the orientation of cations remains fixed toward the inorganic cube. According to previous studies, spin splitting in perovskite is extremely sensitive to the orientation of organic cations and the distortion of inorganic cages [42]. This indicates that the experimental observation of Rashba splitting cannot be attributed to surface reconstruction, and the large Rashba effect is related to the polarity arrangement of MA cations [43].

## CONCLUSION

In summary, we systematically revealed the evolution process of perovskite surfaces under external energy stimulation from the perspectives of element composition, crystal structure, and electronic structure by using surface-sensitive spectroscopic techniques combined with a UHV interconnection system. The detailed degradation pathways on perovskite surfaces are determined by ADXPS and continuously probing X-ray experiments. The phase transition from cubic phase to tetragonal phase on perovskite surfaces under external stimulation is also demonstrated by ARPES combined with DFT calculations. The evolution of electronic structure on perovskite surfaces under external stimulation is also analyzed in detail after surface evolution. As a result of surface decomposition, metallic Pb^0^ acts as donor levels, bending the valence band energy level downward. The experimental value for the effective mass after lattice distortion is determined to be 0.27 *m_0_*, which is between a tetragonal and cubic phase calculated based on DFT. The giant Rashba effect can be observed at low temperatures and is likely due to the polarity arrangement of MA cations. We believe our research helps to understand the origin of the surface properties of perovskite materials and can be applied to perovskite photovoltaic device engineering.

## METHODS

### XPS experiments

The surface composition information was analyzed by ADXPS (PHI 5000 Versaprobe Ⅱ) using monochromatic Al Kα radiation (1486.6 eV), and the X-ray gun with an Al anode runs at 15 KV and 25 W, and the analyzed area is around 0.1 mm in diameter. The ADXPS spectra were characterized by different emission angles from 25° to 65° respecting the sample surface. During our ADXPS experiment, the irradiation spot was moved 0.5 mm to a fresh position after every XPS scan cycle (about 5 minutes). The X-ray aging measurement research was carried out in which the sample was continually exposed to probing X-ray by XPS scanning. An XPS scanning cycle period was set to 6 minutes, and the total scanning time of the continuously probing X-ray experiment lasted for 2 hours. The elemental ratio of the surface is obtained by dividing the core level signal intensity of each element by the respective elemental atomic sensitivity factor.

### ARPES experiments

ARPES measurements were carried out on MAPbBr_3_ single crystals (001) surface using DA30L ARPES SYSTEM equipped with monochromatic He Ⅰα (hν = 21.2 eV) radiation source, where the angular and energy resolutions were set to <0.1° and 20 meV, respectively. The light was focused on the sample surface with spot sizes about 1 mm diameter, and the acceptance angle was covered within ±15°. The system Fermi level (E_F_) was calibrated by using the surface of an Au (111) crystal, while the perovskite single crystals were in electronic contact with the equipment. The sample's temperature ranged from 77 K (liquid nitrogen) to 300 K (room temperature).

### XRD, SEM characterization

The crystalline quality of samples was measured by powder X-ray diffraction (XRD; D8 Advance, Bruker) from 10° to 70° using Cu-Kα radiation. The surface morphology of the as-cleaved samples before and after degradation was characterized by scanning electron microscopy (SEM; Nova NanoSEM450). For the XRD measurement, the samples were measured in ambient atmosphere.

## Supplementary Material

nwae042_Supplemental_File
