# Supplementary material for: The dynamic surface evolution of halide perovskites induced by external energy stimulation
Source: Natl Sci Rev. 2024 Feb 2;11(4):nwae042. doi: 10.1093/nsr/nwae042 (PMC10939416; doi:10.1093/nsr/nwae042)
Supplement: nwae042_Supplemental_File [file nwae042_supplemental_file.pdf]

Supplementary Information

The dynamic surface evolution of halide perovskites induced by external energy stimulation

Feiyu Cheng[1,2,†], Pengdong Wang[3,†], Chenzhe Xu[1,2,†], Qingliang Liao[1,2,*], Suicai Zhang[1,2], Haochun Sun[1,2], Wenqiang Fan[1,2], Guodong Liu[4], Zhiyun Li[3], Yaping Kong[3], Li Wang[3], Fangsen Li[3], Zhuo Kang[1,2,*] and Yue Zhang[1,2,*]

[1] Academy for Advanced Interdisciplinary Science and Technology, State Key Laboratory for Advanced Metals and Materials, University of Science and Technology Beijing, Beijing 100083, China;

[2] Beijing Key Laboratory for Advanced Energy Materials and Technologies, Key Laboratory of Advanced Materials and Devices for Post-Moore Chips, Ministry of Education, Beijing Advanced Innovation Center for Materials Genome Engineering, School of Materials Science and Engineering, University of Science and Technology Beijing, Beijing 100083, China;

[3] Vacuum Interconnected Nanotech Workstation (Nano-X), Suzhou Institute of Nano-Tech and Nano-Bionics, Chinese Academy of Sciences, Suzhou 215123, China;

[4] Beijing National Laboratory for Condensed Matter Physics, Institute of Physics, Chinese Academy of Sciences, Beijing 100190, China

***Corresponding authors.** E-mails: yuezhang@ustb.edu.cn; zhuokang@ustb.edu.cn; liao@ustb.edu.cn

[†]Equally contributed to this work.

Sample preparation

High-quality $MAPbBr_3$ single crystals were grown by the low temperature gradient crystallization (LTGC) method. $MAPbBr_3$ single crystals were cleaved in situ under ultrahigh vacuum conditions. As shown in **Figure 1f**, the samples were adhered to molybdenum sample holders using Torr Seal, and then the ceramic rod was adhered vertically to the top of the sample surface (001) by Torr Seal as well. The silver paste was painted around the sample to ensure electrical conduction between the sample and the sample holder. Then the sample was transferred to the UHV chamber ($<2*10^{-10}$ mbar), and the ceramic rod was hit by a wobble stick. A clean and flat perovskite surface (001) was obtained. The as-cleaved samples in UHV were used for ARPES, ADXPS, and SEM measurements.

DFT

The first principle calculation based on DFT was employed to calculate the electronic structure of cubic and tetragonal perovskite $MAPbBr_3$. The projector augmented wave (PAW) approach implemented in the Vienna ab initio package (VASP) was used to treat the valance electrons described by cut-off plane waves and core electrons described by pseudo wave functions. The Perdew-Burke-Ernzehof (PBE) functional based on generalized gradient approximation (GGA) was used to calculate the exchange-correlation interaction. The cut-off energy of 600 eV and 5×5×5 (cubic)/4×4×3 (tetragonal) Γ-centered *k*-mesh in the Brillouin zone was applied in electronic energy calculation in cell optimization. The crystals were optimized until the self-consistent force is less than 0.01 eV/Å and the energy between two consecutive steps was less than 10^{-7} eV. The electronic structure of crystal perovskite was calculated with cut-off energy of 400 eV and 8×8×8 (cubic)/6×6×4 (tetragonal) Γ-centered *k*-mesh. The K point path was selected as Γ-M-X-R-Γ for the cubic phase and M-Γ-X|R-Z-A for the tetragonal phase in band structure calculation with inserting 20 points between high symmetry K points uniformly. To calculate the effective mass of the hole, the number of the inserting points increased to 50. To calculate the 3D band structure, the interval between the adjacent K points was set as 0.05-0.07 in the Brillouin zone. Tetrahedron integral was used in calculating density of states (DOS). In all calculations, Grimme's dispersion correction (DFT-D3) was applied to describe the weak interaction.

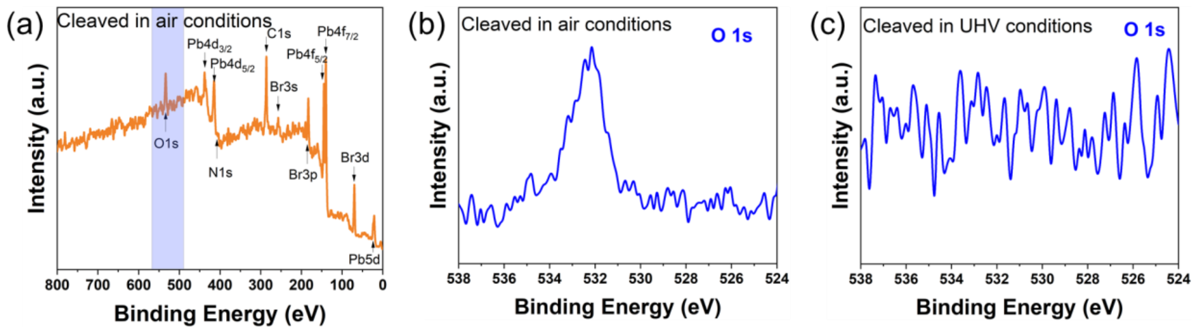

Supplementary Figure S1. (a) Full XPS spectra and (b) O 1s core-level peak of $MAPbBr_3$ surface cleaved in the air atmospheric environment. (c) O 1s core-level peak of $MAPbBr_3$ surface cleaved in UHV conditions

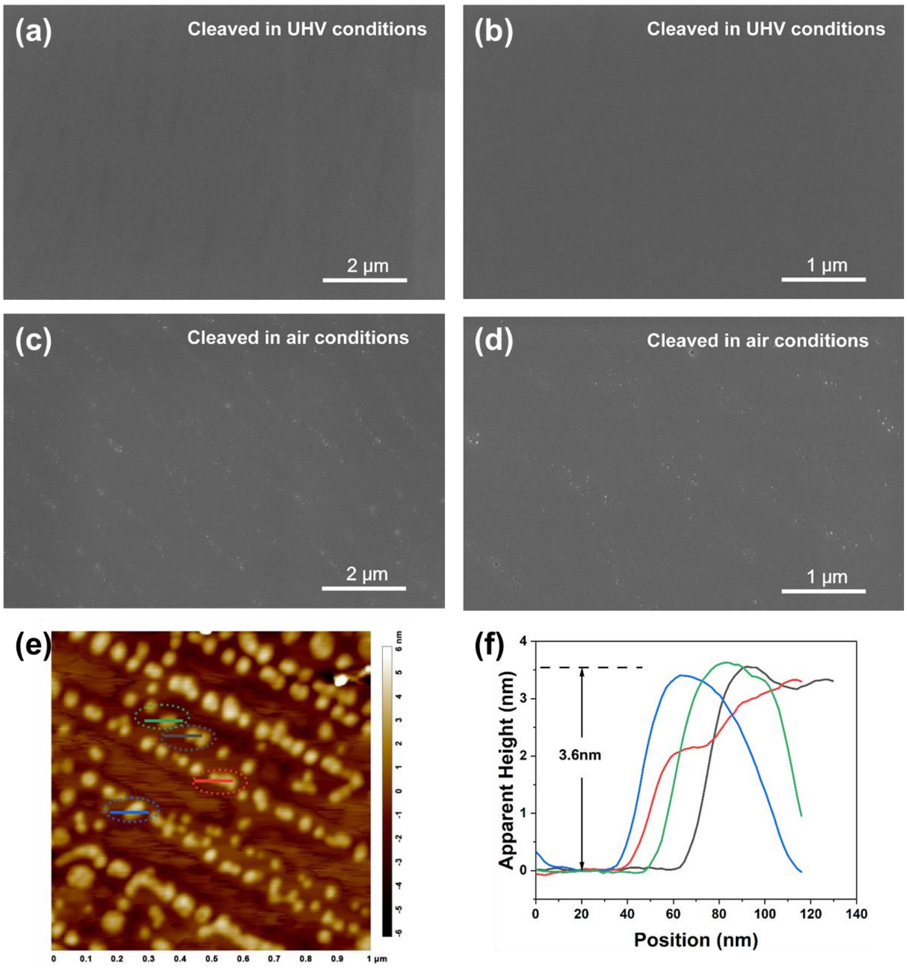

Supplementary Figure S2. The SEM images of the surfaces of (a-b) samples cleaved in situ in a vacuum condition and (c-d) samples cleaved in air condition, respectively. (e)Atomic force microscopy (AFM) image of the surface cleaved in the air condition, (f) line profile on the bright line of perovskite hydrate

protrusions measured along the marked lines in panel (e).

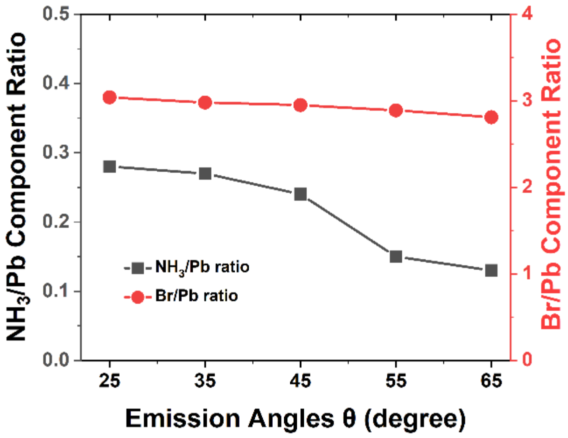

Supplementary Figure S3. Atomic ratios of NH_3/Pb and Br/Pb for the $MAPbBr_3$ surface as a function of the electron emission angle θ determined from ADXPS.

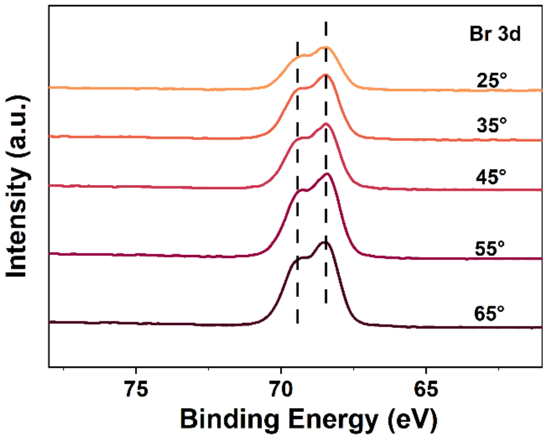

Supplementary Figure S4. ADXPS spectra of Br 3d core levels obtained for pristine $MAPbBr_3$ at different emission angles θ.

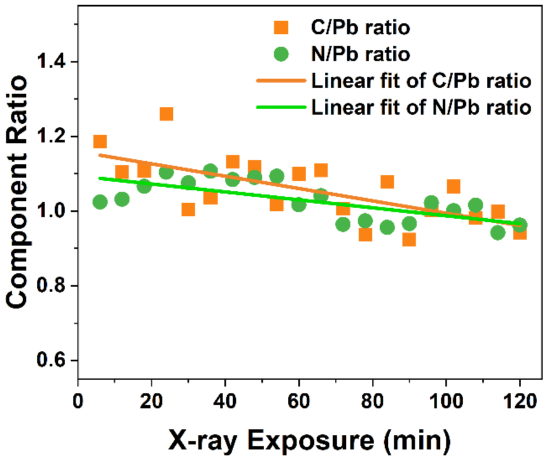

Supplementary Figure S5. Elemental composition C/Pb and N/Pb ratio changes of $MAPbBr_3$ with increasing X-ray irradiation time.

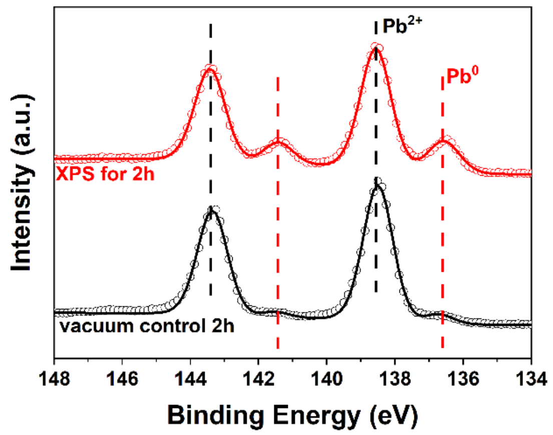

Supplementary Figure S6. The XPS Pb 4f core level spectra of the $MAPbBr_3$ surface under vacuum conditions and X-ray irradiation for 2 h.

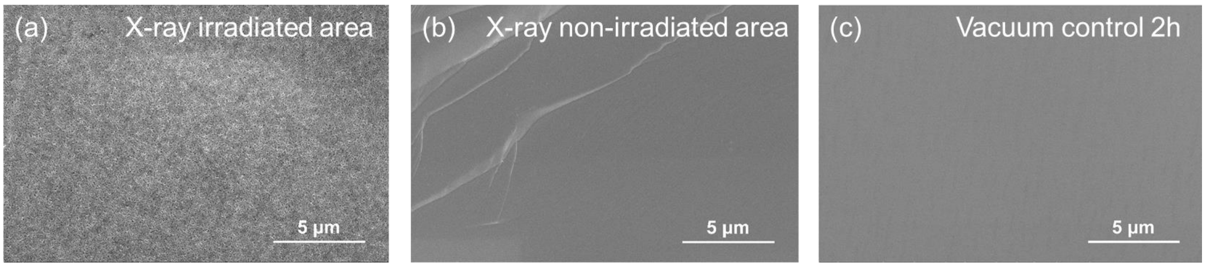

Supplementary Figure S7. SEM images of the sample showing (a) the X-ray irradiated area, (b) the non-irradiated area during X-ray aging measurements, and (c) no X-ray irradiation but vacuum exposure for 2 hours.

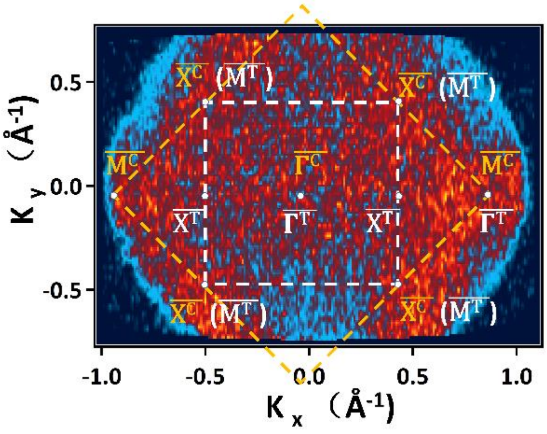

Supplementary Figure S8. The ARPES experimental constant energy cut of the electronic structure at deeper binding energies of crystals $MAPbBr_3$. Cubic and tetragonal surface Brillouin zones are shown in yellow and white, respectively, C and T represent the cubic and tetragonal phase.

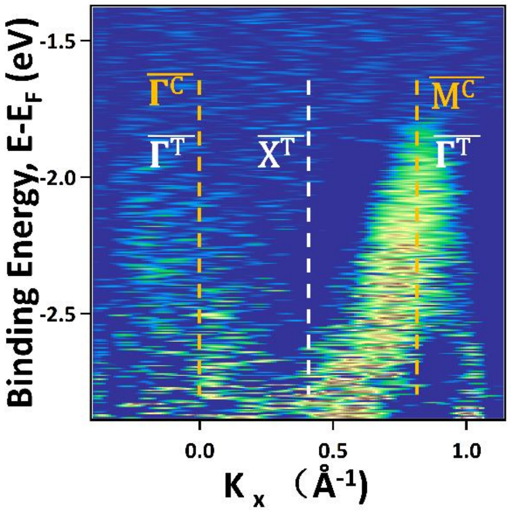

Supplementary Figure S9. ARPES *k*-space second derivative dispersion maps along the cubic ΓM direction.

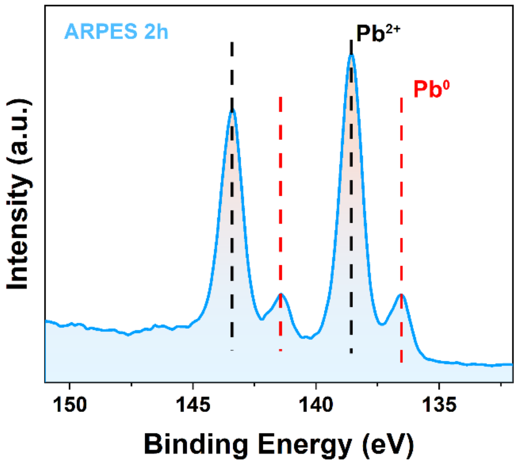

Supplementary Figure S10. Throughout the ARPES measurement, the UV light (21.2 eV by He Ⅰα lamp) is always probing on the sample, and after the ARPES measurements, the samples are immediately transferred to the XPS chamber by the UHV interconnected system. The XPS results are similar to the continuously probing X-ray experiment in **Figure 2d** in the main text.

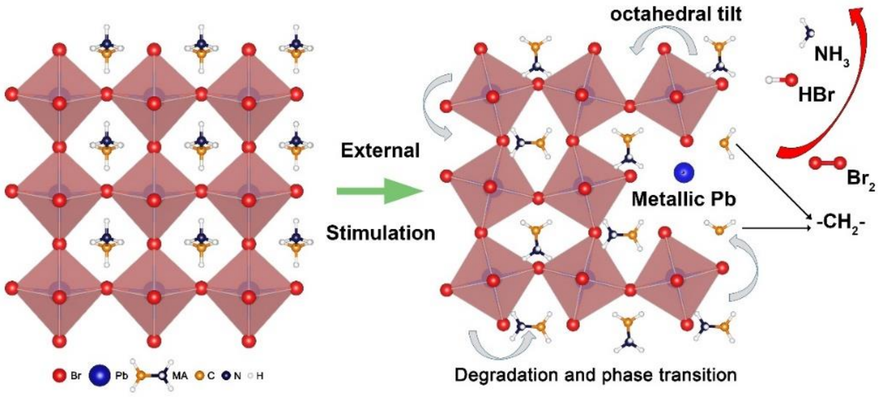

Supplementary Figure S11. Schematic of the external stimulation induced surface degradation process and phase transition for the $MAPbBr_3$ single crystals.

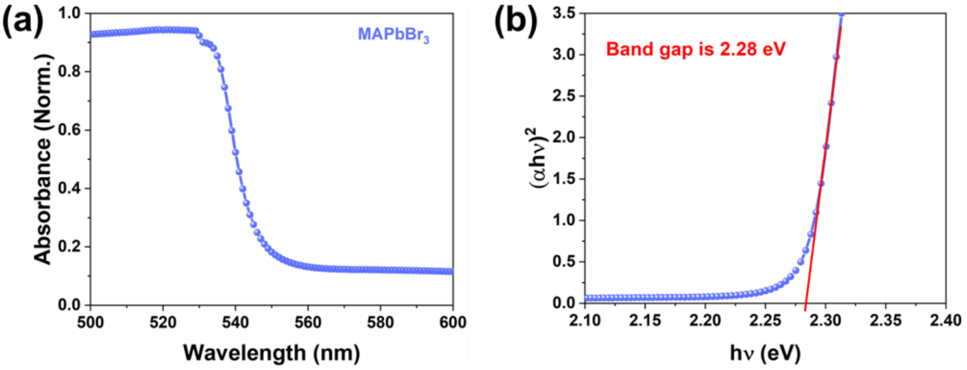

Supplementary Figure S12. (a) Absorption spectra of $MAPbBr_3$ and (b) Tauc plot showing the bandgap (2.28eV).

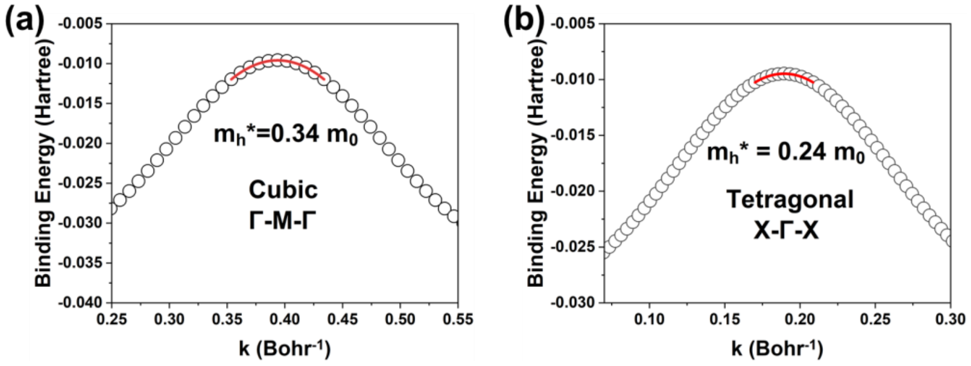

Supplementary Figure S13. Calculated hole effective mass of the $MAPbBr_3$ (a) cubic phase along Γ-M-Γ direction and (b) tetragonal phase along X-Γ-X direction.

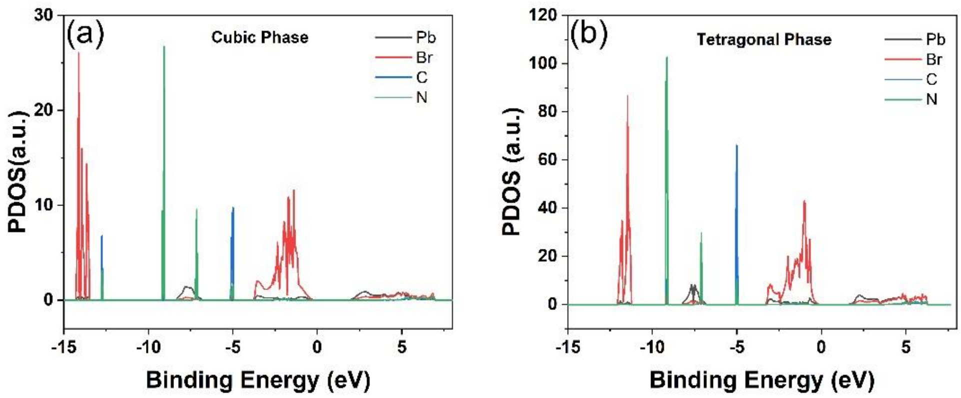

Supplementary Figure S14. Calculated partial density of states (PDOS) of the $MAPbBr_3$ (a) cubic phase

and (b) tetragonal phase.

Supplementary Table S1. The accurate sampling depth of C 1s, N 1s, Pb 4f, Br 3d in $MAPbBr_3$ corresponding to the detection angles from 25 °-65 °.

Elements	Sampling Depth (nm)				
	25 °	35 °	45 °	55 °	65 °
C 1s	3.94	5.35	6.6	7.64	8.46
N 1s	3.65	4.96	6.11	7.08	7.83
Pb 4f	4.32	5.87	7.23	8.38	9.27
Br 3d	4.5	6.11	7.53	8.72	9.65

In XPS measurements, 95% of the information in the no-loss XPS peak comes from the depth of $3\lambda \sin\theta$, which is usually called "sampling depth" (*J. Electron. Spectrosc. Relat. Phenom.* **2009**, 169 (1), 1-9.). The λ value of photoelectron of C 1s, N 1s, Pb 4f, and Br 3d in $MAPbBr_3$ is 3.11 nm, 2.88 nm, 3.41 nm, 3.55 nm respectively, as calculated by TPP-2M method in NIST's (National Institute of Standards and Technology) database. The accurate sampling depth corresponding to the detection angles from 25 °-65 ° is shown in **Supplementary Table S1.**
